# Use of Rgg quorum-sensing machinery to create an innovative recombinant protein expression system in Streptococcus thermophilus

**DOI:** 10.1099/mic.0.001487

**Published:** 2024-09-20

**Authors:** Rozenn Gardan, Edith Honvo-Houeto, Christine Mézange, Nathanael Jean Maillot, Aurélie Balvay, Sylvie Rabot, Luis G. Bermúdez-Humarán, Philippe Langella, Véronique Monnet, Vincent Juillard

**Affiliations:** 1 Paris-Saclay University, INRAE, AgroParisTech, Micalis Institute, Jouy-en-Josas, France

**Keywords:** inducible promoter, heterologous protein production, Rgg, signalling peptide, *Streptococcus thermophilus*

## Abstract

*Streptococcus thermophilus* holds promise as a chassis for producing and secreting heterologous proteins. Used for thousands of years to ferment milk, this species has generally recognized as safe (GRAS) status in the USA and qualified presumption of safety (QPS) status in Europe. In addition, it can be easily genetically modified thanks to its natural competence, and it secretes very few endogenous proteins, which means less downstream processing is needed to purify target proteins, reducing costs. Extracellular degradation of heterologous proteins can be eliminated by introducing mutations that inactivate the genes encoding the bacterium’s three major surface proteases. Here, we constructed an inducible expression system that utilizes a peptide pheromone (SHP_1358_) and a transcriptional regulator (Rgg_1358_) involved in quorum-sensing regulation. We explored the functionality of a complete version of the system, in which the inducer is produced by the bacterium itself, by synthesizing a luciferase reporter protein. This complete version was assessed with bacteria grown in a chemically defined medium but also *in vivo,* in the faeces of germ-free mice. We also tested an incomplete version, in which the inducer had to be added to the culture medium, by synthesizing luciferase and a secreted form of elafin, a human protein with therapeutic properties. Our results show that, in our system, protein production can be modulated by employing different concentrations of the SHP_1358_ inducer or other SHPs with closed amino acid sequences. We also constructed a genetic background in which all system leakiness was eliminated. In conclusion, with this new inducible expression system, we have added to the set of tools currently used to produce secreted proteins in *S. thermophilus*, whose myriad applications include the delivery of therapeutic peptides or proteins.

## Introduction

In the realm of biotechnology, heterologous protein production is most often carried out using *Escherichia coli* or *Bacillus* species as bacterial hosts. However, there is a pressing need to develop other bacterial chassis given that host choice strongly influences potential applications. At present, the two domains of greatest importance are the *in vitro* production of proteins and the *in situ* delivery of proteins with therapeutic properties. A significant hurdle in this work is that it is almost impossible to predict the fate and yield of a given heterologous protein in a specific host, especially when the protein must undergo secretion and/or may experience protease-mediated maturation/degradation [1]. For pharmaceutical applications, lactic acid bacteria (LAB) are of particular interest because they have been granted generally recognized as safe (GRAS) status by the U.S. Food and Drug Administration (FDA) and qualified presumption of safety (QPS) status by the European Food and Safety Authority (EFSA). For example, *Lactococcus lactis* has been successfully used to deliver anti-inflammatory molecules to treat inflammatory bowel disease (IBD) at the mucosal level [2]. However, there are some drawbacks to employing *L. lactis*, including its significant residual levels of cell surface proteolysis; the latter occurs even in strains that lack the major extracellular protease HtrA [3]. Furthermore, only a few strains of *L. lactis* are somewhat naturally competent, which constrains potential genetic modifications [4,5]. Therefore, there is a need to find hosts other than *L. lactis*.

*Streptococcus thermophilus* is one of the main starters used in yoghurt and cheese production and is emerging as a promising cell factory [6], especially for producing heterologous proteins. Its combined traits make it a good candidate host. First, *S. thermophilus* is naturally competent [7,9], allowing for straightforward genetic modifications; it is also a non-sporulating bacterium with a small genome [10]. Second, it can be grown under tightly controlled conditions in milk, rich media or chemically defined media, a trait that could facilitate protein production at different scales. Third, in *S. thermophilus*, surface proteolysis is carried out by three proteases, facilitating the creation of mutant strains in which there is no cell surface proteolysis [11], a property that could be useful when producing secreted proteins. However, to date, *S. thermophilus* has been used to produce just a few heterologous proteins, namely those of a cytoplasmic nature, such as green fluorescent or luciferase reporter proteins [12, 13]. Fourth, because this bacterium remains metabolically active during gastrointestinal transit [14,15], strains that produce bacterial surface-anchored or secreted proteins could be used to (1) induce vaccination by activating the host immune response or (2) promote certain physiological effects in the gastrointestinal tract. As a proof of concept that this species could be used to produce cell-wall-anchored proteins, Lecomte *et al.* successfully expressed the gene encoding the *Lactobacillus helveticus* prtH protease in *S. thermophilus*; no protease activity was detected in the resulting supernatant [16].

To efficiently produce a heterologous protein, the gene encoding the protein must be placed under the control of a specific promoter. Depending on the protein, it may be useful to choose among constitutive promoters (with different strengths) [17] or inducible promoters [12], especially when the protein produced has a negative impact on the growth of the bacterial host. Blomqvist *et al.* built a pheromone-induced expression vector [18] using a bacteriocin production mechanism found in * S. thermophilus.* This quorum-sensing (QS) mechanism, named StbABCDHR, functions with the extracellular detection of the peptide pheromone StbC. Using the *stbD* gene promoter, the researchers confirmed that the system could be induced by the presence of mature synthetic StbC in the extracellular medium. However, the inducible promoter was shown to be leaky (i.e*.* significant levels of expression occurred in the inducer’s absence). Very recently, a tetracycline-inducible promoter was developed in *L. lactis* and was successfully tested in *S. thermophilus* [13]. That said, at present, a limited number of promoters are available and have been tested in *S. thermophilus*.

Other QS mechanisms are present in *S. thermophilus,* and they function based on intracellular pheromone detection. Indeed, pheromones are detected inside cells after exportation, maturation and internalization [19]. Upon arrival in the intracellular environment, pheromones interact with dedicated transcriptional regulators, a process that modifies their activity and, consequently, the expression of target genes. The ComS/ComR system, in which ComS is the pheromone, controls the mechanism that triggers natural competence, which is required for transformation [20,21]. Similarly, SHP/Rgg systems, in which SHP is the pheromone, control different functions including the production of post-translationally modified peptides [22,23]. In this study, we utilized the SHP/Rgg_1358_ system from *S. thermophilus* strain LMD-9 to develop an inducible protein expression system employing the promoter P*_ster1357_*. This choice was based on the fact that, in *S. thermophilus*, promoter-based SHP/Rgg systems, there are only transcription of the SHP encoding gene and the operon found downstream of the *rgg* gene [24]; in contrast, in ComR systems, there is transcription of an extended regulon [25]. We validated the functionality of our inducible expression system by producing a luciferase reporter and a secreted form of elafin, a human protein. We found that our system could be finely controlled by modulating inducer concentrations, and we constructed a genetic background in which all system leakiness was eliminated. Finally, we showed that the system is fully functional *in vivo* – it successfully produced luciferase in the faeces of germ-free mice.

## Methods

### Bacterial strains and growth conditions

**Fig. 1.**
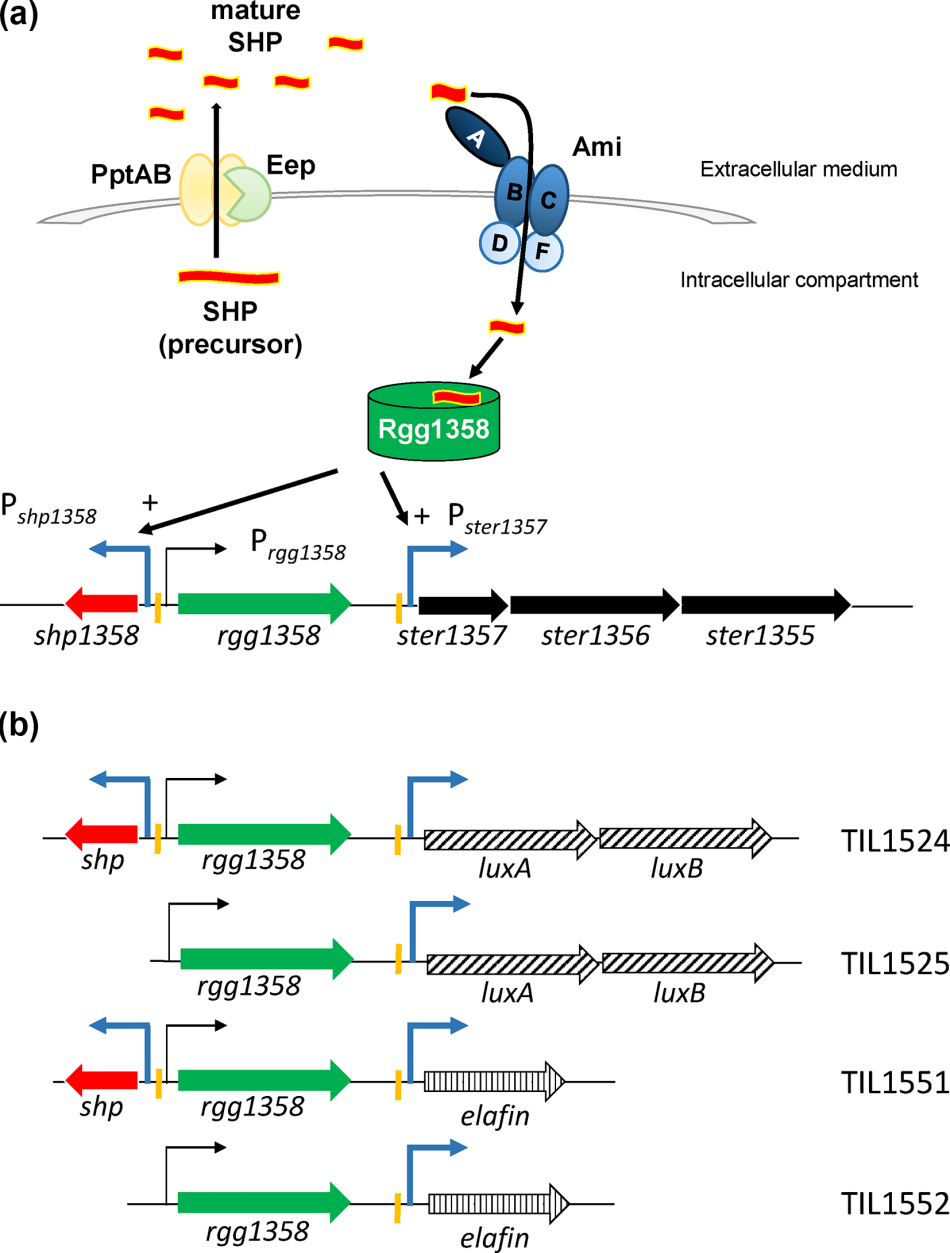
Schematic representation of the SHP/Rgg_1358_ quorum-sensing mechanism in *S. thermophilus* strain LMD-9 and of the genetic constructs used in the study. (**a**) The quorum-sensing signal is a pheromone encoded by the *shp_1358_* gene. SHP_1358_ is a small hydrophobic peptide, whose mature form is produced by the cleavage of a precursor and then exported; the two processes are performed by the endopeptidase Eep and the transporter PptAB, respectively. At high cell densities, secreted SHP_1358_ is reimported into the cell by the Ami transporter and then interacts with the regulatory protein Rgg_1358_. When the resulting SHP/Rgg_1358_ complex binds to the promoter region of the two target genes, P*_shp1358_* and P*_ster1357_*, it activates their transcription. (**b**) DNA constructs encoding the complete or truncated SHP/Rgg_1358_ system were fused to the luciferase-encoding gene (*luxAB*) or the elafin-encoding gene (names of corresponding strains on the right). The solid arrows represent genes (orientation and name underneath). The hatched arrows represent reporter or heterologous protein-encoding genes. The broken arrows represent promoters (name at head of arrow). Blue broken arrows represent promoters regulated by Rgg_1358_.

We used the wild-type (WT) *S. thermophilus* strain LMD-9 [26] as a chromosomal DNA donor to recover the *shp/rgg_1358_* system by PCR (Fig. 1) and the WT strain CNRZ1066 [10] as the host for the different genetic constructions. The *S. thermophilus* strains used in this study are listed in Table 1. *S. thermophilus* strains were grown at 42 °C in either M17 medium (Difco) supplemented with 10 g l^−1^ lactose (M17lac) or in a chemically defined medium (CDM) [27]. *E. coli* strains TG1*repA^+^* [8] or TOP10 (Thermofischer scientific) and *L. lactis* strain MG1363 [28] were used as hosts for intermediary cloning steps. *E. coli* strains were grown in Luria-Bertani (LB) broth with shaking at 30 °C for strain TOP10 and at 37 °C for strain TG1*repA*^+^. *L. lactis* strains were grown at 30 °C in M17 medium (Difco) supplemented with 10 g l^–1^ glucose. Agar (1.5%) was added to the media as needed. When required, antibiotics were added to the media at the following final concentrations: erythromycin at 150 µg ml^−1^ for *E. coli* and at 5 µg ml^−1^ for *S. thermophilus* and *L. lactis*, kanamycin at 1 mg ml^−1^ for *S. thermophilus*, spectinomycin at 150 µg ml^−1^ for *S. thermophilus*, and chloramphenicol at 10 µg ml^−1^ for *E. coli*, *S. thermophilus* and *L. lactis.*

**Table 1.** *Streptococcus thermophilus* strains used in this study

Strain*	Genotype	Resistance†	Description‡	Source or reference
LMD-9	Wild-type	-	-	[26]
CNRZ1066	Wild-type	-	-	[10]
TIL1486	Δ*pptAB::erm*	Erm	-	[24]
TIL1523	Δ*sepM*::*spec*	Spec	PCR fragment *sepM*::*spec* → CNRZ1066	This study
TIL1524	*blp::shp-rgg_1358_-*P*_ster1357_-luxAB-aphA3*	Km	pGICB004a::*shp-rgg_1358_-*P*_ster1357_*→ CNRZ1066	This study
TIL1525	*blp::rgg_1358_-*P*_ster1357_-luxAB-aphA3*	Km	pGICB004a::*rgg_1358_-*P*_ster1357_*→ CNRZ1066	This study
TIL1535	Δ*htrA*::*aphA3*	Km	PCR fragment *htrA*::*aphA3* → CNRZ1066	This study
TIL1536	Δ*htrA*::*aphA3* Δ*sepM*::*spec*	Km Spec	TIL1535 DNA → TIL1523	This study
TIL1551	*blp::shp-rgg_1358_-*P*_ster1357_-elafin-P32cat* Δ*htrA*::*aphA3* Δ*ywdF*::*spec*	Km Spec Cm	pEla:: *shp-rgg_1358_-*P*_ster1357_*→ TIL1536	This study
TIL1552	*blp::rgg_1358_-*P*_ster1357_-elafin- P32cat* Δ*htrA*::*aphA3* Δ*ywdF*::*spec*	Km Spec Cm	pEla::*rgg_1358_-*P*_ster1357_*→ TIL1536	This study
TIL1566	*blp::rgg_1358_-*P*_ster1357_-luxAB-aphA3* Δ*pptAB::erm*	Km	pGICB004a::*rgg_1358_-*P*_ster1357_*→ TIL1486	This study
TIL1567	*blp::rgg_1358_-*P*_ster1357_- elafin- P32cat* Δ*htrA*::*aphA3* Δ*ywdF*::*spec* Δ*pptAB::erm*	Km Erm	TIL1486 DNA → TIL1552	This study
TIL1664	*blp*::P32-*luxAB- aphA3*	Km	pGICB004a::P32→ CNRZ1066	This study
TIL1672	*blp*::*luxAB- aphA3*	Km	pGICB004a→ CNRZ1066	This study

a *All strains TIL strains are derived from strain CNRZ1066.

b†Km, Spec, Cm and Erm: resistance to kanamycin, spectinomycin, chloramphenicol and erythromycin, respectively.

‡The arrows indicate construction via transformation with chromosomal DNA, PCR fragment or a plasmid.

### DNA manipulation and sequencing

Restriction enzymes, T4 DNA ligase (New England Biolabs) and Phusion DNA polymerase (Finnzymes) were used according to the manufacturers’ instructions. Standard methods were used to carry out DNA purification, restriction digestion, PCR, ligation and sequencing. The oligonucleotides used (Eurofins) are listed in Table S1, available in the online version of this article. * S. thermophilus* strain CNRZ1066 or its derivatives were transformed using natural competent cells [8] prepared with the addition of synthetic competence peptide (ComS, LPYFAGCL) at a final concentration of 1 µM. *L. lactis* electrocompetent cells were prepared and transformed as previously described [29]. The plasmids used are listed in Table S2.

### Synthesis of SHP and ComS peptides

The peptide SHP_1358_ (EGIIVIVVG), also named SHP3, was purchased from Chinapep and the peptides ESIIVIAVG (SHP1), EGIIVILVG (SHP4), EGIIVIGVG (SHP5), CIYTIVGGV (SHP8), DIIIIVGG (SHP9) and ComS from Genecust. The purity of the peptides was greater than 95%. SHP peptides were resuspended as 1 mM stock in DMSO for SHPs and water for ComS. Substocks (100 µM) were made in water for both types of peptides before their final dilution and utilization in CDM. All stock solutions were stored at −20 °C.

### Construction of mutant strains

The surface protease mutant strain was obtained on the basis of strain CNRZ1066, in which one of the proteases (PrtS) is naturally lacking, as follows: the overlapping PCR method was used to delete the *sepM* (*STR_RS07745*) and *htrA* (*STR_RS09505*) genes and replace them with a spectinomycin (*spec*) and a kanamycin cassette (*aphA3*), respectively, as previously described [8]. The oligonucleotides are described in Table S1. Briefly, the *spec* and the *aphA3* cassettes were PCR amplified from pAT28 [30] and pKa [31] plasmids, respectively, as DNA template. The upstream and downstream fragments of *sepM* and *htrA* genes were amplified using chromosomal DNA from strain CNRZ1066 as a template. The upstream fragments*,* the cassettes and the downstream fragments were fused by overlapping PCR. The resulting PCR fragments were used to transform strain CNRZ1066, leading to the construction of strain TIL1523 (*sepM::spec*) and strain TIL1535 (*htrA::aphA3*). Strain TIL1536 (*sepM::spec htrA::aphA3*) was constructed by transforming strain TIL1523 with chromosomal DNA from strain TIL1535.

We constructed two versions of our inducible expression system: a complete one working on its own and a truncated one that requires the addition of synthetic SHP in the culture medium to trigger expression. To study the relevance of our inducible expression systems, we used *luxAB* luciferase-encoding genes, and thus two derivatives of plasmid pGICB004a, pGICB004a::*shp-rgg*_1358_-P*_ster1357_* (complete version) and pGICB004a::*rgg*_1358_-P*_ster1357_* (truncated version), were constructed as follows: the *shp-rgg*_1358_-P*_ster1357_* fragment was PCR amplified with oligonucleotides FusLuxCplt-SpeI and FusLux-EcoRI using chromosomal DNA from strain LMD9 as a template; double digested with *Spe*I and *Eco*RI restriction enzymes; and finally ligated into pGICB004a between the related restriction sites leading to the construction of plasmid pGICB004a::*shp-rgg*_1358_-P*_ster1357_* . A similar approach was used for the construction of pGICB004a::*rgg*_1358_-P*_ster1357_* with oligonucleotides FusLuxIncplt-SpeI and FusLux-EcoRI. Both plasmids were linearized by *Sca*I. The linearized plasmid pGICB004a::*shp-rgg*_1358_-P*_ster1357_* was used to transform strain CNRZ1066 leading to strain TIL1524 (*blp::shp-rgg*_1358_-P*_ster1357_-luxAB*) and linearized plasmid pGICB004a::*rgg*_1358_-P*_ster1357_* was used to transform strains CNRZ1066 and TIL1486 (Δ*pptAB::erm*) leading to strains TIL1525 (*blp::rgg*_1358_-P*_ster1357_-luxAB*) and TIL1566 (*blp::rgg*_1358_-P*_ster1357_-luxAB* Δ*pptAB::erm*). To construct strain TIL1664 used as a positive control for the *in vivo* experiment, plasmid pGICB004a::P32 was linearized by *Sca*I and used to transform strain CNRZ1066. To construct strain TIL1672 used as a negative control for the *in vivo* experiment, plasmid pGICB004a was linearized by *Sca*I and used to transform strain CNRZ1066.

To study the functionality of our inducible expression systems, we decided to clone and express a protein of therapeutic interest, elafin [32,33]. For this, we constructed plasmids pEla::*shp-rgg_1358_-*P*_ster1357_* and pEla::*rgg_1358_-*P_ster*1357*_ by replacing the *luxAB* genes and *aphA3* cassette in plasmids pGICB004a::*shp-rgg_1358_-*P*_ster1357_* and pGICB004a::*rgg_1358_-*P*_ster1357_* by the elafin-encoding gene and a chloramphenicol cassette (P32cat), as follows: the elafin gene was PCR amplified with oligonucleotides FusEla-EcoRI_For and FusEla_Rev from plasmid pLB386 (ProbiHôte collection, Micalis Institute, INRAE, unpublished data). The chloramphenicol cassette was amplified with oligonucleotides P32Cat_For and P32Cat-SalI_Rev from plasmid pNZ5319 [34]. The elafin and the chloramphenicol cassette fragments were joined by overlapping PCR, double digested with *Sal*I and *Eco*RI restriction enzymes, and finally ligated into plasmids pGICB004a::*shp-rgg_1358_-*P*_ster1357_* and pGICB004a::*rgg_1358_-*P*_ster1357_* between the related restriction sites leading to the construction of pEla::*shp-rgg_1358_-*P*_ster1357_* and pEla::*rgg_1358_-*P*_ster1357_*. Both plasmids were linearized by *Sca*I and used to transform strain TIL1536 (Δ*htrA::aphA3* Δ*sepM::spec)* leading to strain TIL1551 (*blp::shp-rgg_1358_-*P*_ster1357_-elafin-P32cat* Δ*htrA::aphA3* Δ*ywdF::spec*) and strain TIL1552 (*blp::rgg_1358_-*P*_ster1357_ -luxAB* Δ*htrA::aphA3* Δ*ywdF::spec*). Chromosomal DNA from strain TIL1486 (Δ*pptAB::erm)* was used to transform strain TIL1552 leading to strain TIL1567 (*blp::rgg_1358_-*P*_ster1357_-elafin-P32cat* Δ*htrA::aphA3* Δ*ywdF::spec* Δ*pptAB::erm*).

All constructions were confirmed by PCR and sequenced when necessary.

### Luciferase assays

For kinetic experiments, cells were grown overnight at 42 °C in CDM. These cultures were then diluted in 50 ml of CDM to an optical density at 600 nm (OD_600_) of 0.05 and incubated at 42 °C. Aliquots of 1 ml of cultures were sampled at regular time intervals until the culture reached the stationary phase and analysed as follows: OD_600_ was measured with 1 ml of culture, then 10 µl of a 0.1% nonyl-aldehyde solution was added and luminescence was immediately measured with a Junior LB9509 (Berthold Technologies). Induction of *luxAB-*encoding genes in strain TIL1525 was performed with the addition of SHP_1358_ peptide (EGIIVIVVG) in the growth medium at a final concentration of 1 µM. Similarly, for the luminescence measurement of faeces, 10 µl of a 0.1% nonyl-aldehyde solution was added to 1 ml of a 10^−2^ dilution of the faeces in CDM.

For characterization of the inducibility of the SHP/Rgg_1358_ system with different concentrations of SHP_1358_ peptide or with different SHP peptides, precultures of strain TIL1566 were grown at 37 °C in CDM. The cultures were then diluted to an OD_600_ of 0.05. At the beginning of the cultures, synthetic SHP_1358_ peptide was added at a concentration ranging between 0 and 10 µM and the other SHP synthetic peptides were added at a concentration of 1 µM. Then, 250 µl of the cultures were transferred to the wells of a sterile white microplate covered with a transparent bottom (Greiner). For characterization of the responsiveness of strain TIL1566 toward the inducer during its growth, SHP_1358_ peptide was added every hour for 3 h directly in the wells of the microplate. The OD_600_ and luminescence values of the cultures were monitored at 37 °C every 10 min using an Inﬁnite M200 spectroluminometer (Tecan), as previously described [9]. Results were reported as relative luminescence units divided by OD_600_ (RLU/OD_600_).

**Fig. 2.**
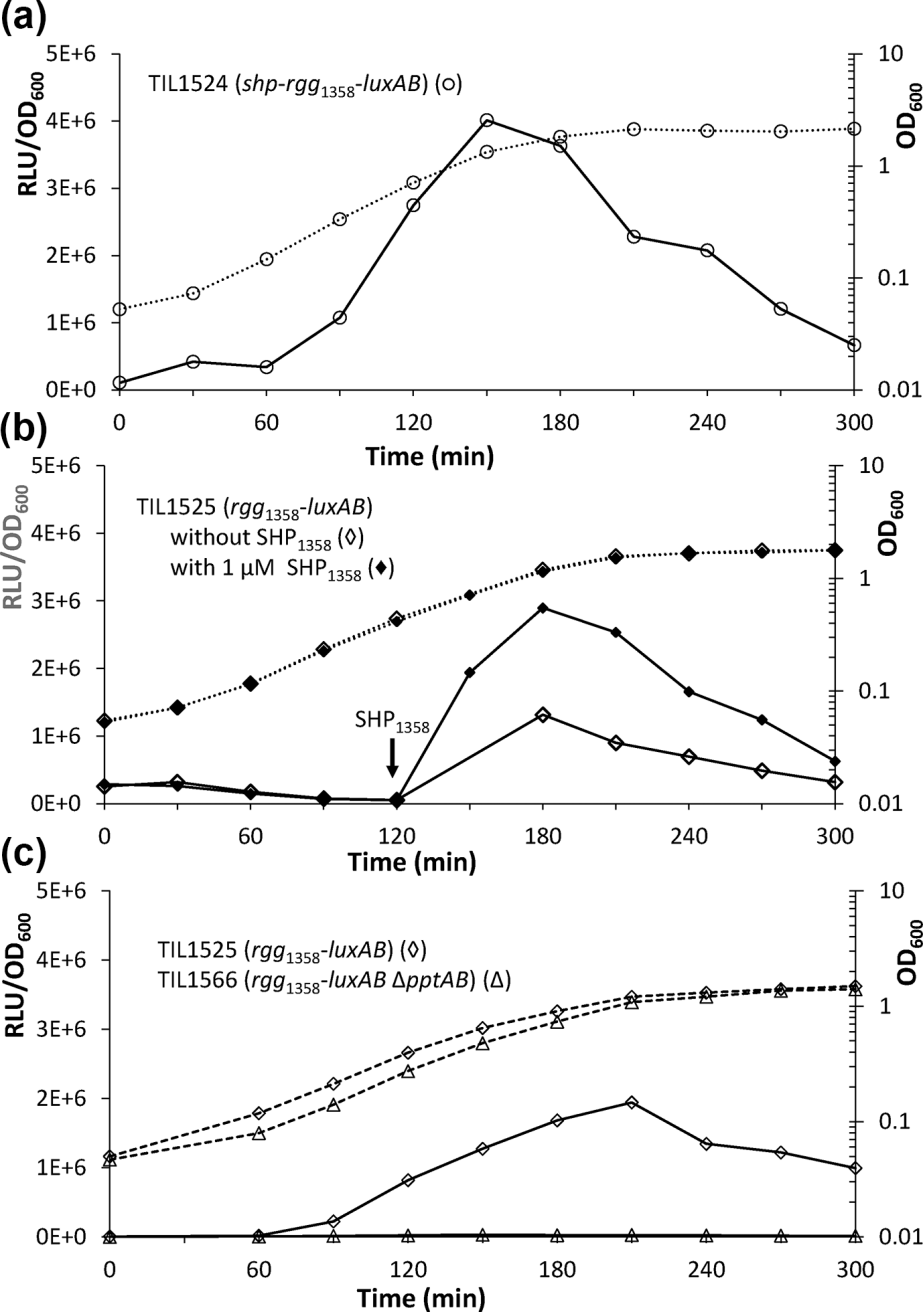
Levels of growth and luciferase luminescence for *S. thermophilus* strains grown under different conditions. (**a**) TIL1524 (*blp::shp-rgg*_1358_-P*_ster1357_-luxAB*) in CDM (◯), (**b**) TIL1525 (*blp::rgg*_1358_-P*_ster1357_-luxAB*) in CDM (◊) or in CDM to which synthetic SHP_1358_ had been added (final concentration of 1 µM) 2 h after the beginning of culture growth (♦), and (**c**) TIL1525 in CDM (◊) and TIL 1566 (*blp::rgg*_1358_-P*_ster1357_-luxAB* Δ*pptAB*) in CDM (Δ). The growth curves (OD_600_) are depicted using dotted lines, and the relative levels of luciferase luminescence (RLU/OD_600_) are depicted using solid lines. Data shown are representative of three independent experiments.

**Fig. 3.**
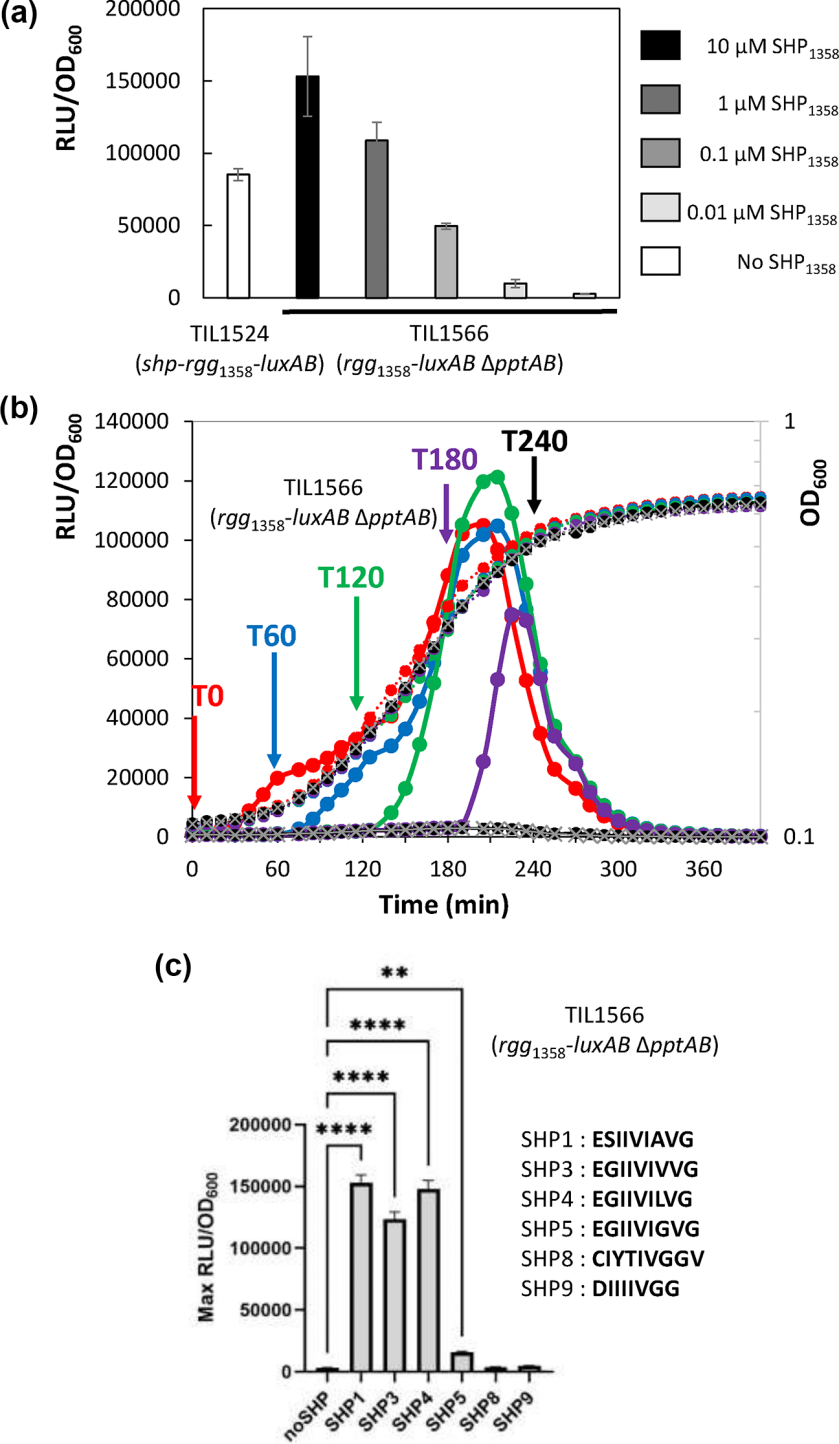
Levels of growth and/or luciferase luminescence for the leakage-free strain, TIL1566 (*blp::rgg_1358_*-P_ster1357_-*luxAB* Δ*pptAB::erm*), grown under different conditions. (**a**) Maximum relative levels of luciferase luminescence (RLU/OD_600_) during strain growth in CDM across a range of SHP_1358_ (SHP3) concentrations (0–10 µM). Strain TIL1524 (*blp::shp-rgg*_1358_-P*_ster1357_-luxAB*) was used as a control. The data shown are the means±sd of the results from four independent experiments. (**b**) Strain growth (OD_600_) and luciferase luminescence (RLU/OD_600_) in CDM to which SHP_1358_ was added at different time points; the coloured curves (•) show the results of SHP_1358_ being added at different time points, and the grey curves (×) show the results of the situations where no SHP_1358_ was added. The growth curves are depicted using dotted lines, and the relative levels of luciferase luminescence are depicted using solid lines. Data shown are representative of three independent experiments. (**c**) Maximum relative levels of luciferase luminescence (RLU/OD_600_) during strain growth in CDM to which different SHPs had been added (1 µM). The data are the means±sd of the results from three independent experiments. To test for significant differences between the treatments and the control, one-way ANOVAs were performed, followed by Dunnett’s tests for multiple comparisons (**P<0.01; ****P<0.0001).

### Western blot assays and elastase tests

For sample preparation of the Western blot assays, derivatives of strain CNRZ1066 were grown in CDM at 42 °C. When necessary, induction was performed by the addition of SHP_1358_ peptide (EGIIVIVVG) at a final concentration of 0.5 µM in the growth medium at an OD_600_ of 0.2. When the cultures reached the desired OD_600_ (depending on the experiment), 10 ml was centrifuged at 3200 ***g*** for 10 min at room temperature. The filtered supernatants (0.22 µM, Millex GV PVDF, Millipore) were further ultrafiltered through Amicon devices [Ultra-15 (3 kDA cutoff); Millipore] for 1 h at 25 °C at 3200 ***g***. A volume of 500 µl containing the secreted elafin was recovered in the upper compartment of the Amicon device. Then, 10 µl of the samples was diluted in Laemmli Buffer (4×) and heated to 95 °C for 5 min. Samples were run on Precast Bis-Tris Gel 4–12% (NuPAGE 1.5 mm×10; Invitrogen) and transferred to nitrocellulose membranes (Trans–Blot turbo 0.2 µm PVDF; Bio-Rad). The membranes were blocked for 2 h at room temperature in a TBST solution (0.1 M Tris pH 7.5, 1.5 M NaCl, 1% Tween 20) containing 5% skimmed milk and then hybridized for 2 h at room temperature in TBST solution with anti-elafin antibodies (Santa Cruz sc-398075, 1:1000 dilution). Four washing steps were performed in TBST solution, one for 15 min and three for 5 min, and detection was achieved using secondary antibody coupled to peroxidase (Abliance BI2413C, 1:1000 dilution) for 1 h. Four washing steps were again performed as described above before the addition of a solution containing luminol (ECL prime; GE healthcare) for 5 min. Five microlitres of recombinant elafin (RD system, 1:100 dilution) was used as a control. Detection was finally recorded using a Chemidoc imaging system (Bio-Rad).

For sample preparation of the elastase assays (EnzChek elastase assay kit; Thermo Fisher Scientific), strains were grown in CDM until they reached an OD_600_ of 1. Supernatants were recovered via centrifugation (at 10 000 r.p.m. at 4 °C for 10 min), filtrated over a 0.22 µm filter (Millipore) and then ultrafiltered successively through 10 and 3 kDa cut-off Amicon filters in order to concentrate the supernatants 200 times. The elastase assays were then performed in accordance with the manufacturer’s instructions. Elastase activity was detected based on the fluorescence reading obtained at 485/530 nm (excitation/emission) from a Biotek SynergyMx microplate reader.

### *In vivo* assays

#### Animals

Nine male and nine female C3H/HeN germ-free mice, 10–12 weeks old, were obtained from the germ-free rodent breeding unit of Anaxem (the germ-free animal facility of the Micalis Institute, INRAE, France). They were transferred into three flexible-film isolators (Getinge), which were ventilated with HEPA-filtered sterile air under positive pressure. The isolators were fitted with a DPTE aseptic transfer system (Getinge) allowing sterile connection of containers (Getinge) to import sterile consumables and germ-free mice. Inside each isolator, three male and three female mice were housed separately in collective cages (three mice per cage) containing sterile bedding made of wood shavings. The living environment was enriched with sterile shredding paper and gnawing wood sticks. The mice had free access to autoclaved tap water and a γ-irradiated (45 kGy) standard diet (R03; Scientific Animal Food and Engineering). The animal room was maintained at 20–24°C and kept on a 12 h light/dark cycle. The mice were examined daily to ensure they stayed healthy.

#### Design of animal studies

The mice were individually identified with electronic chips (IntelliBio) implanted under the skin in the interscapular region. As adaptive responses of *S. thermophilus* in the gut may be improved by lactose [35], this sugar was added to the drinking water (4.5%, w/v) throughout the experiment. On day 0 (D0), mice from a given isolator were inoculated intragastrically, using flexible gavage tubes, with 0.2 ml of a cell suspension of one of the following three strains: TIL1664 (*blp*::P32-*luxAB- aphA3*) (1.44×10^8^ c.f.u. ml^−1^); TIL1524 (*blp::shp-rgg_1358_-*P*_ster1357_-luxAB-aphA3*) (5.15×10^8^ c.f.u. ml^−1^); TIL 1672 (*blp::luxAB- aphA3*) (5.2×10^9^ c.f.u. ml^−1^). Then, fresh faeces were collected from each mouse on D2–3, D7, D10 and D14 to monitor the establishment of the strain and the luciferase activity produced by the bacterial cells. The last day (D15), the mice were killed by cervical dislocation. Colonization of the three strains in the gastrointestinal tract was assessed by counts obtained by plating on M17lac plates dilutions of faeces (in CDM for 10^−1^ and 10^−2^ dilutions and in 0.1% tryptone for the following dilutions). The 10^−2^ dilution was used for a manual luciferase assay as described above.

## Results

### Choice of the SHP/Rgg_1358_ system for heterologous protein production in *S. thermophilus*

The *S. thermophilus* pangenome encodes at least nine SHP/Rgg systems and one stand-alone Rgg system (V. Juillard, unpublished data). On average, *S. thermophilus* strains contain five *shp*/*rgg* pairs; such is the case for the strains used in this study, LMD-9, which is a common model in genetic studies of this species, and CNRZ1066. When an RNA sequencing approach was applied to strain LMD-9, it highlighted the presence of two Rgg transcriptional regulators, Ster_RS06405 and Ster_RS06695, whose target genes were expressed at high levels when the QS system was functional and at low levels when the QS system was disrupted [24]. These target genes are located downstream of the two Rgg-encoding genes. Given the relative genetic organization of the *shp* and *rgg* genes, we decided to focus on the gene encoding Ster_RS06695 because the position of its locus facilitates the construction of genetic scaffolding for heterologous protein production systems. In contrast, at the locus containing the Ster_RS06405-encoding gene, the genes encoding Rgg and SHP are convergent and overlapping. The STER_RS06695 gene is hereafter designed as *rgg*_1358_ based on a primary annotation as Ster_1358 in NCBI [26] and the use of this annotation in previous publications. The SHP gene (hereafter, SHP_1358_ or SHP3) is not annotated in the NCBI database. This gene encodes a 23 aa peptide (MKKQILLTLLLVVFEGIIVIVVG), for which maturation occurs in front of the glutamate residue, releasing a 9 aa peptide (sequence underlined above). The maturation process is carried out by the membrane protease Eep [36], and secretion into the extracellular medium is performed by the transporter PptAB [24]. The mature peptide (also called the autoinducing peptide) is then imported by the Ami oligopeptide permease. Once inside the cell, the mature SHP_1358_ peptide can interact with the Rgg_1358_ peptide to positively control the expression of its target genes (1) *shp_1358_*, creating a positive feedback loop, and (2) a polycistronic operon whose first three genes are involved in the production and secretion of a cyclic peptide called streptide [36,38] (Fig. 1a). The first gene of the operon, *ster_1357*, encodes the precursor of streptide. When the *shp_1358_* gene has been eliminated from the system, the addition of synthetic mature SHP_1358_ to the culture medium can restore *shp_1358_* expression in a dose-dependent manner [36]. The DNA sequence recognized by the SHP/Rgg_1358_ complex has been identified in the promoter region of the *shp_1358_* gene and of the *ster_1357* gene [36]. In conclusion, we chose to use the SHP/Rgg_1358_ complex in combination with the promoter of the streptide polycistronic operon (P*_ster1357_*) to construct an inducible expression system in an *S. thermophilus* strain that naturally lacks this system: CNRZ1066 [10].

### Validation of the SHP/Rgg1358 inducible expression system in *S. thermophilus* strain LMD-9 using luciferase as a reporter

When validating the functionality of our SHP/Rgg_1358_ inducible expression system, we utilized luciferase as a reporter protein because it is straightforward to quantify luciferase luminescence. We took advantage of the natural competence of *S. thermophilus* to introduce all the constructs into the chromosome of each experimental strain. This localization led to greater recombinant stability and precluded the need for maintenance via antibiotics. Two forms of the system were assessed (Fig. 1b). The first form of the system consisted of the *shp_1358_* gene, the *rgg*_1358_ gene and the promoter of the streptide operon, P*_ster1357_*, and was used to generate strain TIL1524 (*blp::shp-rgg*_1358_-P*_ster1357_-luxAB*). This version of the system was complete (i.e*.* self-inducible) and was expected to trigger the expression of the target gene under the control of the P*_ster1357_* promoter during the mid-exponential growth phase. The second form of the system lacked the *shp_1358_* gene and was used to generate strain TIL1525 (*blp::rgg*_1358_-P*_ster1357_-luxAB*). This version of the system was incomplete (i.e. truncated or inducible) since target gene expression required the addition of mature synthetic SHP_1358_ to the growth medium. As expected, when strain TIL1524 (*blp::shp-rgg*_1358_-P*_ster1357_-luxAB*) was grown in CDM, luciferase luminescence appeared during the mid-exponential growth phase, with relative levels peaking at the end of exponential growth (3.7±0.3×10^6^ RLU/OD_600_; Fig. 2a). Strain TIL1525 (*blp::rgg*_1358_-P*_ster1357_-luxAB*) was grown in two different media: CDM and CDM with synthetic SHP_1358_ (Fig. 2b). When grown in the CDM with synthetic SHP_1358_, the strain rapidly displayed luciferase luminescence, with relative levels peaking at the end of the exponential growth phase (3.2±0.3×10^6^ RLU/OD_600_). However, even in the CDM without SHP_1358_, there was a significant relative level of luciferase luminescence (1.5±0.2×10^6^ RLU/OD_600_). Although strain CNRZ1066 naturally lacks an SHP/Rgg_1358_ system, its genome does encode five other SHP/Rgg systems that produce mature SHPs, some of them with sequences highly similar to those of SHP_1358_ (Table S3). It seems likely that, in strain TIL1525, these endogenous SHPs were interacting with heterologous Rgg_1358_ and thereby causing luciferase expression. To test this hypothesis, we introduced the reporter fusion of strain TIL1525 (*blp::rgg*_1358_-P*_ster1357_-luxAB*) into strain TIL1486 (Δ*pptAB::erm*). In the resulting strain, TIL1566 (*blp::rgg*_1358_-P*_ster1357_-luxAB* Δ*pptAB::erm*), none of these endogenous SHPs could be exported, as confirmed by the complete absence of luciferase luminescence (Fig. 2c). These results indicate that the promoter leakiness or cross-induction by endogenous SHPs that was occurring in strain TIL1525 could be entirely eliminated by the introduction of the Δ*pptAB* construct. Also, as expected, when SHP_1358_ was added to the culture medium for the leakage-free strain (TIL1566), we were able to restore luciferase expression, as detailed below.

### Induction of the SHP/Rgg_1358_ expression system is dose dependent

An important sign that an inducible expression system is functioning properly is that the expression of the genes under promoter control can be modulated by inducer concentration. Thus, to assess the functionality of our SHP/Rgg_1358_ system, we grew strain TIL1566 (*blp::rgg*_1358_-P*_ster1357_-luxAB* Δ*pptAB::erm*) in CDM to which different concentrations of SHP_1358_ had been added. In this experiment and the other luciferase production experiments described below, small-volume cultures (250 µl) were grown in microplates. We were able to tightly control maximum relative levels of luciferase luminescence between SHP_1358_ concentrations of 0 and 1 µM (Fig. 3a). Within this range, we did not observe any clear impacts of inducer concentration on strain growth (Fig. S1). At 10 µM, relative levels of luciferase luminescence exceeded those obtained with the self-inducible system (strain TIL1524), but variability among the biological replicates was also higher.

Another important sign of system functionality is that the expression of the genes under promoter control can be triggered rapidly at different time points during growth. Using strain TIL1566 (*blp::rgg*_1358_-P*_ster1357_-luxAB* Δ*pptAB::erm*), we conducted an experiment in which the treatment groups received SHP_1358_ at a final concentration of 1 µM at a given 60 min interval during culture growth (T=0, 60, 120, 180 or 240 min). When the addition of SHP_1358_ occurred at 0, 60 or 120 min, luciferase luminescence appeared immediately, and its levels rapidly increased (Fig. 3b). For these three treatment groups, maximum relative levels of luciferase luminescence were similar in magnitude at the end of the exponential growth phase. When the addition of SHP_1358_ occurred at 180 min, the maximum relative level of luciferase luminescence was lower, and, when it occurred at 240 min, no induction was seen. Unfortunately, it is impossible to interpret levels of luciferase luminescence after the late exponential growth phase because levels of FMNH2, luciferase’s cofactor, have declined, leading to luciferase inactivity [39].

### Different SHPs can activate the SHP/Rgg1358 inducible expression system

As noted above, the presence of luminescence during growth by the strain with the truncated system (TIL1525: *blp::rgg*_1358_-P*_ster1357_-luxAB*) suggests that strain CNRZ1066 produces endogenous SHPs that can interact with Rgg_1358_. We thus conducted an experiment to test this hypothesis, to evaluate the efficacy of these potential endogenous SHPs as system inducers, and to assess whether target genes could be modulated by inducers other than SHP_1358_. The amino acid sequence of mature SHPs encoded by the genome of strain CNRZ1066 is described in Table S3. We thus grew the leakage-free strain with the truncated system (TIL1566: *blp::rgg*_1358_-P*_ster1357_-luxAB* Δ*pptAB::erm*) in CDM containing mature synthetic forms of these SHPs versus in CDM containing mature synthetic SHP_1358_ (SHP3) and then determined the maximum relative levels of luciferase luminescence. We found that three of these peptides had effects: SHP1, SHP4 and SHP5 (Fig. 3c). Maximum relative levels of luciferase luminescence were similar in response to SHP1 and SHP4 as they had been in response to SHP_1358_. SHP5 was less effective.

### The SHP/Rgg1358 inducible expression system is functional in the gut of germ-free mice

**Fig. 4.**
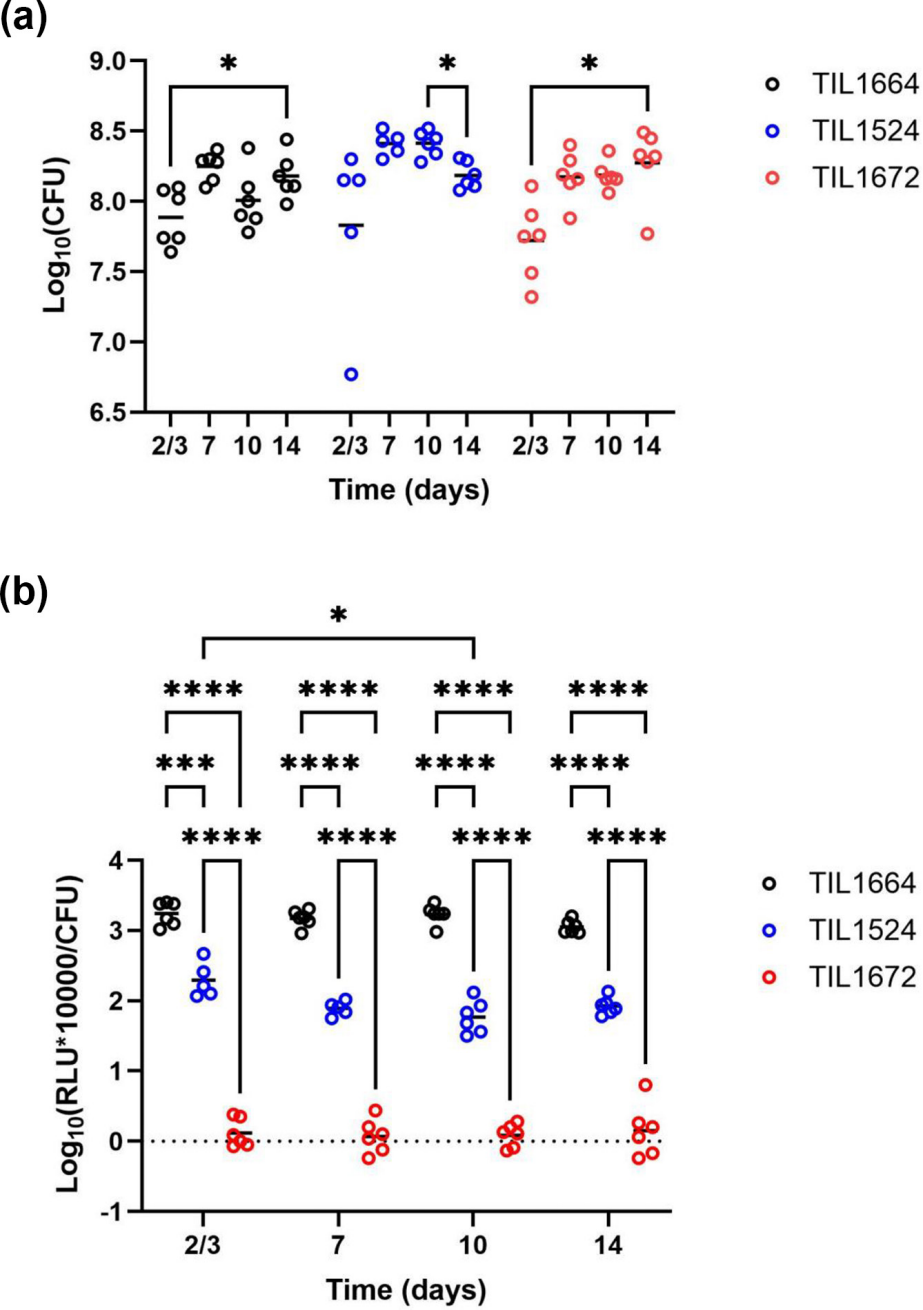
Bacteria abundance and luciferase luminescence in the faeces of germ-free mice that had been inoculated with *S. thermophilus* strain TIL1664 (*blp*::P32-*luxAB-aphA3*) (black open circles), TIL1524 (*blp::shp-rgg_1358_*-P_ster1357_-*luxAB-aphA3*) (blue open circles) and TIL 1672 (*blp::luxAB- aphA3*) (red open circles). Faeces were sampled on days 2–3, 7, 10 and 14. (**a**) Counts (log_10_ c.f.u. g^−1^ faeces) were obtained by diluting and plating the faeces on M17lac. (**b**) Relative levels of luciferase luminescence (log_10_ RLU×10000 c.f.u. g^−1^ faeces) were measured using a 10^−2^ dilution of the faeces immediately after their dilution in CDM. To test for significant differences among treatments, repeated-measures two-way ANOVAs were conducted, followed by Tukey’s tests for multiple comparisons (*P<0.05; ***P<0.001; and ****P<0.0001).

Given its GRAS and QPS statuses, *S. thermophilus* is a good candidate for the *in situ* delivery of therapeutic molecules (e.g*.* peptides or proteins). To test the potential utility of our SHP/Rgg_1358_ inducible expression system in this context, we conducted an experiment using the strain with the complete system (TIL1524: *blp::shp-rgg*-P*_ster1357_-luxAB*). Strain TIL1664 (*blp*::P32-*luxAB*) was used as a positive control; its luciferase-encoding genes (*luxAB*) were under the control of P32, a strong constitutive promoter. The negative control was strain TIL1672 (*blp::luxAB*), whose luciferase-encoding genes were not under the control of a dedicated promoter. We chose to work with germ-free mice, whose gastrointestinal tracts are readily colonized by *S. thermophilus* (10^8^ c.f.u. g^−1^ faeces) as long as lactose is added to the drinking water [40]. Germ-free mice were taken from a given isolator and inoculated intragastrically with one of the three strains. Their faeces was recovered four times over the course of 14 days to monitor bacteria abundance and relative levels of luciferase luminescence. The bacteria reached expected levels of abundance starting on day 7 [>8 log_10_ (c.f.u. g^−1^ faeces)] (Fig. 4a). At each sampling point, there were no significant differences in bacteria abundance among strains, but there were significant differences in the strains’ relative levels of luciferase luminescence (Fig. 4b). Luciferase luminescence levels were nearly null for the negative control (strain TIL1672); they reached around 3 log_10_ (RLU×10 000 c.f.u.^−1^) for the positive control (strain TIL1664). For the strain with the self-inducible system (TIL1524), luciferase luminescence was around 2 log_10_ (RLU×10 000 c.f.u.^−1^), indicating that the expression system was functioning *in vivo*, albeit at lower levels than those achieved with the strong constitutive promoter.

### The SHP/Rgg1358 inducible expression system can produce elafin

**Fig. 5.**
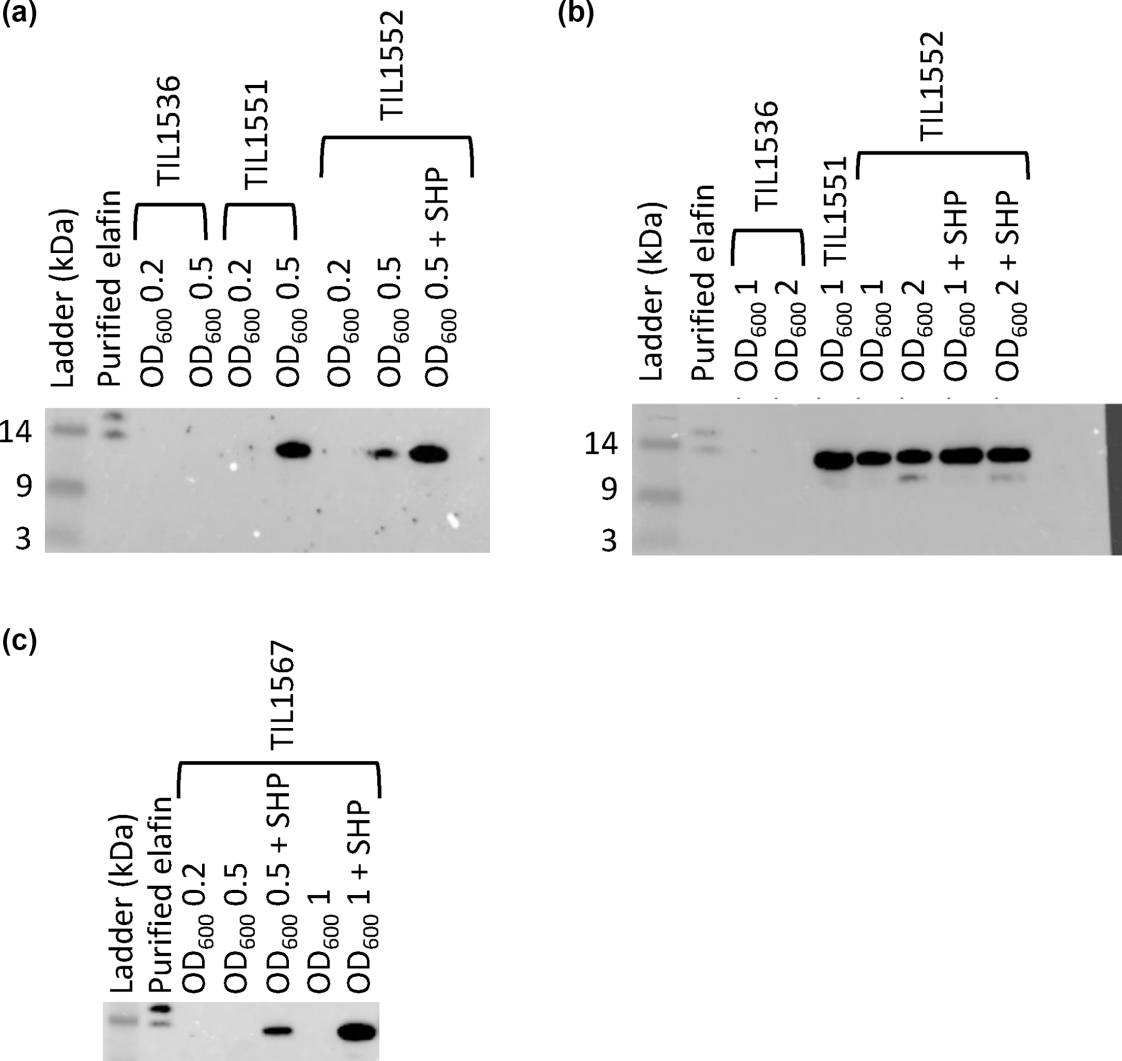
Presence of elafin in the supernatant of different *S. thermophilus* strains as detected by Western blot. (**a**) At OD_600_=0.2 and 0.5 for strain TIL1536 (Δ*htrA::aphA3* Δ*sepM::spec*), TIL1551 (*blp::shp-rgg_1358_-*P*_ster1357_-elafin-P32cat* Δ*htrA::aphA3* Δ*ywdF::spec*) and TIL1552 (*blp::rgg_1358_-*P*_ster1357_-elafin-P32cat* Δ*htrA::aphA3* Δ*ywdF::spec*). (**b**) At OD_600_=1 and 2 for strain TIL1536, TIL1551 and TIL1552. (**c**) At OD_600_=0.2, 0.5 and 1 for strain TIL1567 (*blp::rgg_1358_-*P*_ster1357_-luxAB-aphA3* Δ*htrA::aphA3* Δ*ywdF::spec* Δ*pptAB::erm*). Purified elafin was the positive control, and supernatant from strain TIL1536 was the negative control (i.e*.* the strain does not produce elafin). Elafin production was induced by adding synthetic SHP (+SHP) to the culture medium at OD_600_=0.2.

Elafin is an protease inhibitor found in the human gut and is known to display a protective effect against IBD [33,41]. We thus tested the functionality of our inducible expression system by determining whether it could produce elafin. The *luxAB* genes and the *aphA3* kanamycin cassette of the two plasmids pGICB004a::*shp-rgg*_1358_-P*_ster1357_* and pGICB004a::*rgg*_1358_-P*_ster1357_* were replaced by the elafin-encoding gene with upstream a DNA fragment encoding the secretion signal peptide (SP) of USP45 protein, the main secreted protein of *L. lactis* [42] and downstream a P32Cat cassette conferring resistance to chloramphenicol. Both of the resulting plasmids were linearized and used to transform strain TIL1536 (*ΔhtrA::aphA3* Δ*sepM::spec*). This strain lacks the two major extracellular proteases found in *S. thermophilus* strain CNRZ1066 and displays reduced surface proteolytic activity, which should limit the degradation of secreted heterologous proteins [11]. Two strains were obtained: TIL1551, which had the complete form of the system (*blp::shp-rgg_1358_-*P*_ster1357_-elafin-P32cat* Δ*htrA::aphA3* Δ*ywdF::spec*), and TIL1552, which had the truncated form of the system (*blp::rgg_1358_-*P*_ster1357_-elafin-P32cat* Δ*htrA::aphA3* Δ*ywdF::spec*) (Fig. 1b). Next, both strains were grown in CDM. In the case of strain TIL1552, elafin expression was induced by adding SHP_1358_ at an OD_600_ of 0.2. Supernatant samples were recovered at different OD_600_ values, and the presence of elafin was determined using Western blot analysis. For the strain with the complete system (TIL1551), elafin was not detected at OD_600_=0.2 but was detected at OD_600_=0.5 (Fig. 5a) and OD_600_=1 (Fig. 5b). For the strain with the truncated system (TIL1552), when no SHP_1358_ was added, elafin was not detected at OD_600_= 0.2, started to be detected at OD_600_=0.5 (Fig. 5a) and was clearly detected at OD_600_=1 and 2 (Fig. 5b). At OD_600_=2, a faint band corresponding to an elafin degradation product was also seen . When SHP_1358_ was added, elafin levels at OD_600_=0.5 were much greater than in the absence of SHP_1358_ (Fig. 5a). This induction-related difference was less pronounced at OD_600_=1 and 2 (Fig. 5b). These results clearly highlight that induction was effective at OD_600_=0.5 during the mid-exponential growth phase, while also confirming the leakiness associated with the inducible promoter [i.e*.* in the absence of SHP_1358_, elafin was nonetheless present at higher OD_600_ values (1 and 2)]. To overcome this drawback, we introduced a Δ*pptAB::erm* construct into strain TIL1552, creating strain TIL1567 (*blp::rgg_1358_-*P*_ster1357_-elafin-P32cat* Δ*htrA::aphA3* Δ*ywdF::spec* Δ*pptAB::erm*). When this new strain was used, elafin was no longer detected at either OD_600_=0.5 or 1 in the absence of SHP_1358_ (Fig. 5c). However, as expected, the presence of elafin in the extracellular medium was restored when SHP_1358_ was added. We used strain TIL1536 as the negative control because it does not contain the elafin-encoding gene in its genome. As expected, no traces of elafin were found in any samples for this strain.

**Fig. 6.**
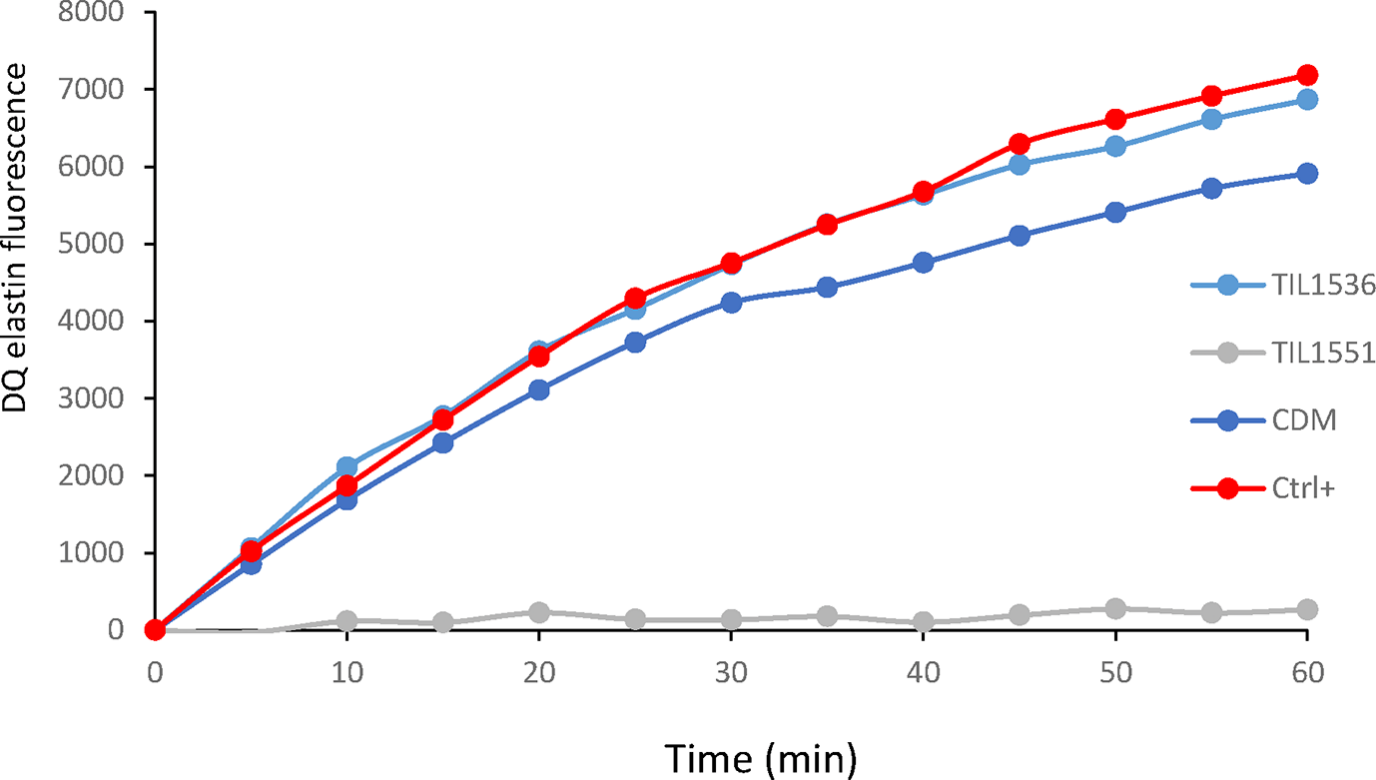
Elastase activity inhibition of the supernatant of strain TIL1551(*blp::shp-rgg_1358_-*P*_ster1357_-elafin-P32cat* Δ*htrA::aphA3* Δ*ywdF::spec*) measured using an EnzChek elastase assay kit. Supernatants of strain TIL1536 (Δ*htrA::aphA3* Δ*ywdF::spec*) and CDM were used as negative control. Ctrl+, positive control of the kit only containing the elastase enzyme and the fluorescent substrate, DQ elastin. Data shown are representative of three independent experiments.

Finally, we decided to determine whether the elafin produced and secreted by *S. thermophilus* was biologically active. To this end, we used a porcine pancreatic elastase assay, since elafin is a specific inhibitor of the elastase. Strain 1551 (*blp::shp-rgg_1358_-*P*_ster1357_-elafin-P32cat* Δ*htrA::aphA3* Δ*ywdF::spec*) was grown in CDM and supernatant was recovered at OD_600_=1 and concentrated 200 times. As a negative control, we used supernatant from strain 1536 (Δ*htrA::aphA3* Δ*ywdF::spec*), which did not produce any elafin. As shown on Fig. 6, the concentrated supernatant of strain 1551 inhibited totally the activity of the elastase whereas the concentrated supernatant of strain 1 536 had no inhibitory effect. This result confirmed that strain TIL1551 secretes a biologically active elafin that is able to inhibit the activity of elastase.

## Discussion

We have developed a new bacterial host chassis for producing high-quality heterologous proteins by introducing a QS system into *S. thermophilus* strain CNRZ1066.

First, we validated the functionality of a complete form of the system, which contained the gene encoding the SHP_1358_ pheromone, the gene encoding the Rgg_1358_ transcriptional regulator and the inducible P*_ster1357_* promoter of the streptide operon. This system was used to produce two active compounds: an intracellular form of luciferase (origin: *Photorabdus luminescens*), whose luminescence is easy to detect during culture growth, and elafin, a secreted human protein with therapeutic properties. One advantage of our system is that it is self-inducible: heterologous protein production occurred immediately and rapidly during the mid-exponential growth phase under laboratory conditions. We also validated the functionality of our system *in vivo*: it successfully produced luciferase in the gut of germ-free mice, which indicates that it could potentially be used to deliver pharmaceuticals. These results are consistent with those of a previous study using R-IVET technology, in which researchers determined that two genes in the operon controlled by the P*_ster1357_* promoter were specifically expressed under simulated conditions of human digestion [43]. The next step will be to assess levels of luciferase expression in the gut of mice with natural or humanized microbiota. Another advantage of our system is that it is localized in the chromosome, which ensures recombinant stability over time and precludes the need for maintenance via antibiotics; this contrasts with the situation of recombinant plasmids [44]. Indeed, levels of luciferase expression *in vivo* remained stable over a 14 day period.

Second, we explored the use of a truncated form of the system, which lacked the gene encoding the SHP_1358_ pheromone. Thus, its functionality was dependent on the addition of synthetic SHP_1358_ to the culture medium. We used this inducible version of the system to again produce luciferase and elafin. SHPs are easy to synthesize because they are linear nonapeptides with no post-translational modifications. The advantage of the system’s inducible form is that it does not impact the host bacterium’s physiology, as long as all nutritional requirements (e.g*.* amino acids, nitrogen or carbon) are met by the growth medium. We did detect leakiness – target protein expression in the absence of added SHP_1358_ – which we were able to completely eliminate via the inactivation of the *pptAB* genes. In *S. thermophilus*, the PptAB oligopeptide transporter seems to be devoted to exporting SHP-type pheromones and ComS, the competence peptide [24]. Surprisingly, we found that two peptides encoded by the genome of strain CNRZ1066 were as efficient as SHP_1358_ when used as synthetic inducers. It seems quite likely that at least one of these peptides is produced by strain CNRZ1066 and was thus responsible for cross-activating the inducible promoter. Interestingly, a third potential SHP of strain CNRZ1066, with an amino acid sequence close to the sequences of the two efficient SHPs, appeared to induce the system at low levels. Further work is needed to understand these differences in efficiency, such as affinity measurements between Rgg and SHPs. Thus, SHPs other than SHP_1358_ could be synthesized and then used to modulate heterologous protein production, depending on specific system applications. These findings also suggest that it may be possible to refine modulation by optimizing the sequence of the inducer, such as by using a synthetic peptide library. Finally, it would be interesting to explore the production of other heterologous proteins, especially those that could have a negative effect on *S. thermophilus* physiology and for which, therefore, the truncated system could prove particularly useful [45]. Indeed, during bacterial growth, we observed an immediate and rapid increase in luciferase production following the addition of the inducer, which indicates that growth (cell density) can be decoupled from protein production.

The SHP/Rgg_1358_ system is absent from approximately 50% of *S. thermophilus* strains. Therefore, it should be possible to introduce the complete or truncated system into the chromosome of one or more of these strains. Such could take place as in this study: the construct was introduced at the non-functional bacteriocin *stb* locus of *S. thermophilus* strain CNRZ1066 [46]. Alternative introduction sites include previously tested permissive loci, such as the tRNA serine and *suc* loci [47], or non-functional ORFs [48]. It is worth noting that, because some strains do not have any SHP/Rgg systems, cross-activation would be unlikely, making it unnecessary to delete the *pptAB* genes. For the remaining 50% of strains that naturally carry the SHP/Rgg_1358_ system, it would be possible to replace the streptide operon with a gene of interest at the homologous locus. This task could easily be accomplished using the Golden Gate Assembly technique, as long as naturally competent strains were used [13].

Functionality of the complete or truncated system is highly dependent on medium composition. In this regard, our system has a major advantage. First, the system was functional during growth in CDM devoid of peptides [49]. Second, the host bacterium, *S. thermophilus*, is a species that releases very few proteins and peptides into the extracellular medium during growth [11]. As a result of these two features, far less downstream processing would be needed to extract heterologous proteins from the extracellular medium, reducing downstream processing costs. In rich media, there are qualitative and quantitative effects of peptide composition, as illustrated by the fact that, for two different yeast extracts, there were two different levels of expression for two genes under the control of the streptide operon located downstream of *ster1357* [50]. As their name suggests, these media are richer and make it possible to reach higher cell densities, which are also likely to result in higher protein yields.

In conclusion, our *S. thermophilus* chassis was not designed to compete with classical chassis (*E. coli* or *B. subtilis*) in terms of heterologous protein yields. However, this inducible expression system, which can be used in strains in which cell surface proteases have been inactivated, makes it possible to produce secreted proteins that will experience little to no degradation. More generally, this system adds to the existing suite of LAB chassis. It also exploits a bacterium that is naturally competent, thereby facilitating the introduction of genetic modifications. The dual GRAS–QPS status of *S. thermophilus* also means it can be used in certain targeted applications.

## supplementary material

10.1099/mic.0.001487 Uncited Supplementary Material 1.

## Abbreviations

CDM: chemically defined medium
cfu: colony forming unit
GRAS: generally recognized as safe
IBD: inflammatory bowel disease
LAB: lactic acid bacteria
QPS: qualified presumption of safety
QS: quorum sensing
RLU: relative luminescence units
SD: standard deviation
WT: wild type
