# Supplementary material for: Use of Rgg quorum-sensing machinery to create an innovative recombinant protein expression system in Streptococcus thermophilus
Source: Microbiology (Reading). 2024 Sep 20;170(9):001487. doi: 10.1099/mic.0.001487 (PMC11414475; doi:10.1099/mic.0.001487)
Supplement: Uncited Supplementary Material 1. [file mic-170-01487-s001.pdf]

Table S1

Oligonucleotides used in this study.

Primer	Sequence (5' → 3')[a,b]
Amplification of the spectinomycin cassette	
Spec_For	GATCTGTATAATAAAGAATA
Spec_Rev	AGCCTAATTGAGAGAAGTTTC
Amplification of the kanamycin cassette	
aphA3_For	CCAGCGAACCATTTGAG
aphA3_Rev	GTTGCGGATGTACTTCAG
Amplification of the chloramphenicol cassette	
P32Cat_For	TCCTCGGGATATGATAAGATTAATAG
P32Cat-SalI_Rev	GAAGAAGTCGACTCTCATATTATAAAAGCCAGTCATTAG
Amplification of upstream and downstream fragments of the *sepM* gene	
Up_sepM_For	TTCGAGGCCTACGCAATGCG
Up_sepM_Rev	TACAGATTAATAATTATTCTTTATTATACAGATCCAGAGTAATTTCCAGTTGCC
Down_sepM_For	GAAAAATTCTATAGAAACTTCTCTCAATTAGGCTAAGGCTGATCCGGATGCCAA
Down_sepM_Rev	CCCAACAACACCAGGCTCATT
Amplification of upstream and downstream fragments of the *htrA* gene	
Up_htrA_For	GTAATCACGGTCACCAACC
Up_htrA _Rev	GACATCTAATCTTTTCTGAAGTACATCCGCAACAGTAAACCACCTAGTAAGCC
Down_htrA _For	ATAATCTTACCTATCACCTCAAATGGTTCGCTGGGTAGTGTTCAGAAAGGTATGCC
Down_htrA _Rev	GGATTGAGATTTGATCGTTG
Construction of plasmid pGICB004a::*shp-rgg*$_{1358}$-P$_{ster1357}$	
FusLuxCplt-SpeI	ACCTACTAGTATGCCGTTGCTTTTTGG
FusLux-EcoR1	GAAGAATTCTCTTTAGACATATTTGGTAC
Construction of plasmid pGICB004a::*rgg*$_{1358}$-P$_{ster1357}$	
FusLuxIncplt-SpeI	ACCTACTAGTAATCCTCACTTTTGTTTTTC
Construction of plasmids pEla:: *shp-rgg*$_{1358}$-P$_{ster1357}$ and pEla::*rgg*$_{1358}$-P$_{ster1357}$	
FusEla-EcoRI_For	GAAGAATTCAATGGAGGAAAGTATATGAA
FusEla_Rev	CTATTAATCTTATCATATCCCGAGGAACTAGTCCTCACTGGGGAA

[a]The recognition sequences for the restriction enzymes are underlined with solid lines.
[b]The inverse and complementary sequences for the spectinomycin, kanamycin or chloramphenicol cassettes are underlined with short dashes.

Table S2

Plasmids used in this study.

Plasmid	Description[a]	Source or reference
pGICB004a	Erm,Km, pG⁺host9 derivative containing the *luxAB* genes of *Photorhabdus luminescens* and an kanamycin resistance cassette surrounded by two fragments of the *blp* operon, allowing double crossover integration at the *blp* locus of *S. thermophilus*	(Fleuchot et al., 2013)
pGICB004a::*shp*-*rgg*$_{1358}$-P$_{ster1357}$	Erm, Km, pGICB004a derivative used to introduce a *shp*-*rgg*$_{1358}$-P$_{ster1357}$-*luxAB* transcriptional fusion at the *blp* locus of *S. thermophilus*	This study
pGICB004a::*rgg*$_{1358}$-P$_{ster1357}$	Erm, Km, pGICB004a derivative used to introduce a *rgg*$_{1358}$-P$_{ster1357}$-*luxAB* transcriptional fusion at the *blp* locus of *S. thermophilus*	This study
pGICB004a::P32	Erm, Km, pGICB004a derivative used to introduce a *P32*-*luxAB* transcriptional fusion at the *blp* locus of *S. thermophilus*	This study
pEla:: *shp*-*rgg*$_{1358}$-P$_{ster1357}$	Erm, Cm, pGICB004a derivative used to introduce a *shp*-*rgg*$_{1358}$-P$_{ster1357}$-*elafin* transcriptional fusion at the *blp* locus of *S. thermophilus*	This study
pEla::*rgg*$_{1358}$-P$_{ster1357}$	Erm, Cm, pGICB004a derivative used to introduce a *rgg*$_{1358}$-P$_{ster1357}$-*elafin* transcriptional fusion at the *blp* locus of *S. thermophilus*	This study

[a]Erm, Km and Cm = resistance to erythromycin, kanamycin and chloramphenicol, respectively.

Fleuchot, B., Guillot, A., Mézange, C., Besset, C., Chambellon, E., Monnet, V., and Gardan, R. (2013). Rgg-associated SHP signaling peptides mediate cross-talk in streptococci. PLoS One 8, e66042.

Table S3

Amino acid sequences of the SHP found in *S. thermophilus* strain CNRZ1066. The amino acid sequence of the mature form of the SHP_{1358} (SHP3) LMD-9 is **EGIIVIVVG**.

Locus tag of the *rgg* gene/ SHP tag	Amino acid sequence of the corresponding SHP*
STR_RS07375/ SHP4	MKKQKLLLLVVLVC**EGIIVILVG**
STR_RS09145/SHP5	MNKKALFSLLFVIL**EGIIVIGVG**
STR_RS04530/SHP1	MNKESFLAILLLIF**ESIIVIAVG**
STR_RS01100/SHP8	MKLLKIIVLLT**CIYTIVGGV**
STR_RS05050/SHP9	MEKVSKILPILILVM**DIIIIVGG**

* the predicted sequence of the mature form is in bold

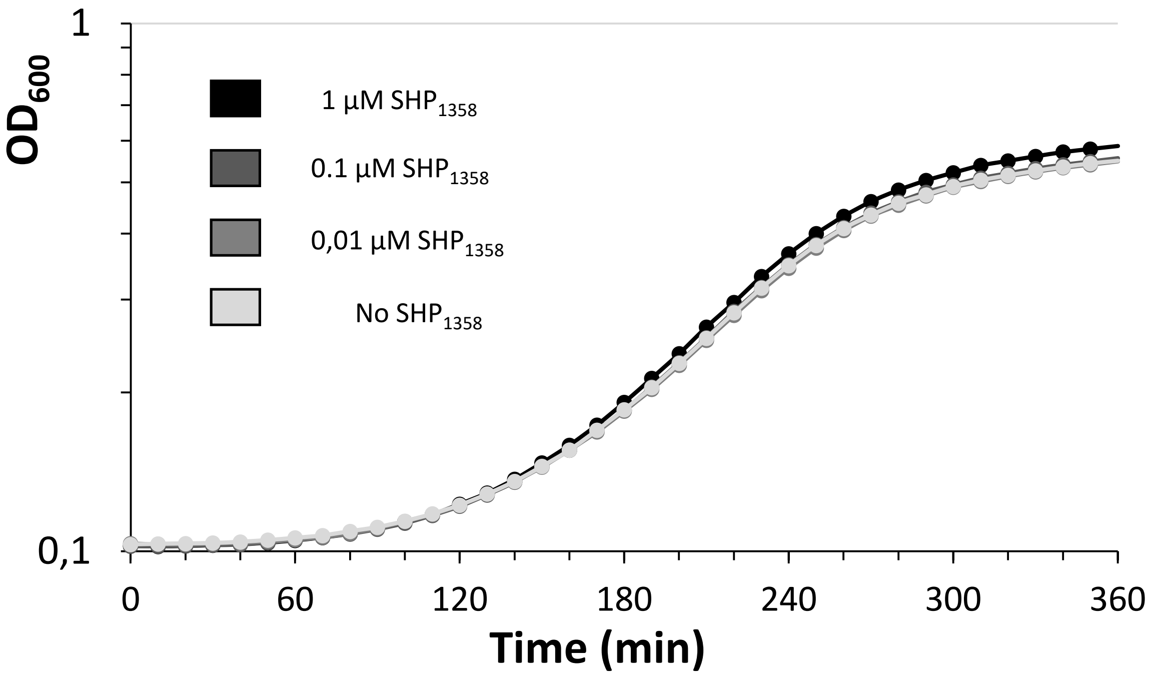

Supplementary Figure 1. Growth of the reporter strain TIL1566 (*blp*::rgg$_{1358}$-P$_{ster1357}$-*luxAB* Δ*pptAB*::*erm*) in CDM with increasing concentration of SHP$_{1358}$ as depicted on the figure. Data shown are representative of four independent experiments.
